# Process Behavior of Short Glass Fiber Filled Systems during Powder Bed Fusion and Its Effect on Part Dimensions

**DOI:** 10.3390/polym13183144

**Published:** 2021-09-17

**Authors:** Lydia Lanzl, Dietmar Drummer

**Affiliations:** 1 Institute of Polymer Technology, Friedrich-Alexander-Universität (FAU) Erlangen-Nürnberg, Am Weichselgarten 9, D-91058 Erlangen, Germany; lkt-info@fau.de; 2 Collaborative Research Center 814—Additive Manufacturing, Friedrich-Alexander-Universität (FAU), Am Weichselgarten 9, D-91058 Erlangen, Germany

**Keywords:** powder bed fusion, filled systems, PA 12, short glass fibers, additive manufacturing, absorption

## Abstract

In powder bed fusion of polymers, filled systems can provide a wide range of part properties, which is still a deficit in additive manufacturing, as the material variety is limited. Glass fiber filled polymers provide a higher strength and stiffness in parts; nevertheless, the process behavior differs from neat polymer systems. In this study, the optical properties and their effect on the part dimensions are analyzed. A higher glass fiber content leads to an increased absorption of laser energy, while the specific heat capacity decreases. This results in larger part dimensions due to higher energy input into the powder bed. The aim of the study is to gain process understanding in terms of ongoing mechanisms during processing filled systems on the one hand and to derive strategies for filled polymer systems in powder bed fusion on the other hand.

## 1. Introduction

Powder bed fusion of polymers (PBF/P) is an additive manufacturing method which allows the generation of parts with a highly complex geometry and filigree structures. While the technology has been established in rapid prototyping over the last years, PBF/P is now on the road to small batch production [1] and is expanding to more and more fields of application. One challenge is the necessity for more materials to grow the potential of additive manufacturing in some market niches [2]. The PBF/P process consists of three sub-processes, which are powder recoating, energy input, coalescence and cooling [3], all which have to be taken into account for process optimization or developing new materials. By using filled systems in PBF/P, the mechanical properties can be tailored and therefore the application range for this process is expanded. Polymeric composites are of high scientific interest due to their potential for functional applications in, for example, aerospace, automobile and marine industries [4]. Using short glass fibers as fillers can lead to an increased stiffness while offering the potential to strengthen a material at the same time, which is interesting for housing applications. Nevertheless, the process behavior, like flowability, packing porosity or optical behavior, is changed by using fillers depending on their type, size distribution and shape [5]. The interaction between these sizes is the deciding factor of whether a powder is suitable for the PBF/P-process. Furthermore, the process parameters might be adapted according to the powder properties. Using a CO_2_-laser, the PBF/P-process is an optical process making the optical properties of the powders an important factor [6]. Depending on the energy input and on the filler content, the part properties change as the fillers have an effect on the optical and thermal properties. In this study, the diffuse reflection and specific heat capacity of powder composites with increasing glass fiber content were analyzed and their effect on part dimensions is determined. Moreover, three different energy densities were used to process the parts to derive process optimization for glass fiber filled systems. Based on these experiments, a deeper understanding on the mechanisms of glass filled systems during processing can be made in which the dependency of energy density and filler content is shown.

## 2. State of the Art

Former studies show that glass beads have an effect on the optical properties as they lead to a decreased diffuse reflection [7] and therefore a higher absorption. Whether this knowledge can be transferred to other shapes, more precisely to fibers having a high aspect ratio, will be considered in this study.

Analyzing filled systems in PBF/P is mainly focused on the effect of fillers on mechanical properties as these are the deciding factors for applications. Nevertheless, it is essential to consider the cause-effect relationships on the process intermediate sizes. A short glass fiber filled poly (butylene) terephthalate (PBT) system was analyzed concerning the resulting powder and part properties by Arai et al. [8]. A higher part porosity and a decreasing shrinkage with increasing filler content could be detected. In another study [9], the effect of anisotropy in the build direction and laser-scanning conditions on the parts was observed, and it was determined that a double-scan method improved mechanical and thermal properties as well as that the shrinkage differs depending on single- and double-scan scanning.

Brecher et al. [10] showed that CO_2_ laser radiation is absorbed by more than 95% in a PP-GF70 prepreg and stated that the radiation is not only absorbed by the thermoplastic material but also by the glass fibers. A typical penetration depth of CO_2_ laser radiation in solid polymers is specified with 100 µm [11]. Nevertheless, the absorption of laser light into powders is higher than into solid material of the same composition [12]. First results showed that the radiation is mainly absorbed by the first glass fiber layers and only partly by the pure thermoplastic layer at the surface [10]. The transmittance of pure glass at a wavelength of 10.6 µm is very low, and therefore heating occurs primarily at the surface and is then conducted through the substrate. With increasing temperature, the transmittance of glass is decreased [13]. Childs et al. [14] investigated polyamide 12 and glass-filled polyamide 11 experimentally and by simulation, and concluded that the laser beam heats the glass powder in its path to a higher temperature than the polymer powder, which leads to a local superficial melting and adhesion of the polymer on to the glass. Conduction leads then to an equalization of temperature. Further studies on glass filled systems [15,16,17] are about the effect of fillers on mechanical properties, but do not mainly treat the process behavior. The temperature-dependent behavior of polyamide 12 was investigated by Laumer et al. [18], who concluded that at a wavelength of 1.94 µm (thulium fibre laser), the optical behavior is not changed until the process temperature of 168 °C. Raising the temperature up to 200 °C, the transmittance increases steeply and the reflectance and absorption decreases due to the melting of the powder. Crystalline areas and therefore the multiple reflections are reduced, and Cui et al. [19] is confirmed, who stated that the crystalline areas have the main influence on the absorptance.

While the state of the art presents some single aspects of optical or thermal behavior of glass filled polymers, the effects during the process of PBF/P and its boundary conditions are still unknown. While most publications on filled systems deal with mechanical properties, a fundamental analysis on the process behavior and resulting effects on part dimensions has hardly been conducted so far. It is important to analyze the transfer from powder to process to part behavior.

This study aims to treat the effect of glass fiber content and energy density on glass fiber filled polyamide 12 systems to derive a more profound process understanding.

## 3. Materials and Methods

### 3.1. Materials

For investigating the effect of the concentration of short glass fibers on polyamide 12, PA 2200 by EOS GmbH and glass fibers by ECTA Handelsgesellschaft mBH are used. The data of the matrix material are depicted in Table 1 and of the filler in Table 2, respectively.

### 3.2. Methodology

#### 3.2.1. Composite Dry-Blending and Processing

**Figure 1:**
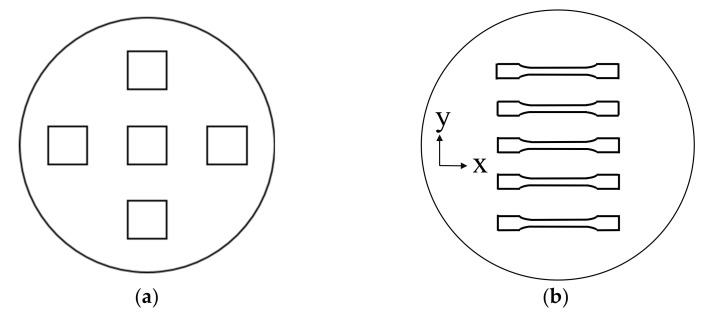
Top view of the build job layouts: (**a**) build job layout for single layers, (**b**) build job layout for parts.

The different concentrations of short glass fibers (10, 30, 40 and 50 vol.%) were dry-blended with the PA 12 powder and then mixed in a turbular mixer by Somakon for 30 min at 300 rpm. For selective beam melting, the powder was processed in a DTM Sinterstation 2000. The processing parameters are listed in Table 3. Three different energy densities were used, while only the laser power was varied and the other parameters were kept constant, to get a basic understanding of the absorption behavior of glass fiber filled systems in PBF/P. This range of the processing parameters were set similar to these of neat PA 12, where an energy density of 0.04 J/mm^2^ is the standard energy density. Values of 0.03 and 0.06 J/mm^2^ depict the lowest and highest applicable energy density for this material. Five tensile bars (DIN EN ISO 3167, Type A) and three single layers per energy density were generated. Two single layer build jobs were carried out. The parameters were statistically distributed over the build job to eliminate position dependencies. A top view of the build job layout is shown in Figure 1.

#### 3.2.2. Powder Analysis

##### DRIFTS (Diffuse Reflection Fourier Transformation Infrared Spectroscopy)

Optical behavior of powder systems was determined by analyzing the diffuse reflection via DRIFTS. An infrared spectrometer (Nicolet 6700, Thermo Scientific, Waltham, MA, USA) and a DRIFTS unit (DiffusIR, PIKE Technologies) in an observed wave number range of 400–4000 cm^−1^ were used. The diffuse reflection at the wave number 943 cm^−1^, which equals the wave length of 10.6 µm of the CO_2_-laser, was then analyzed.

##### Specific Heat Capacity

**Figure 2:**
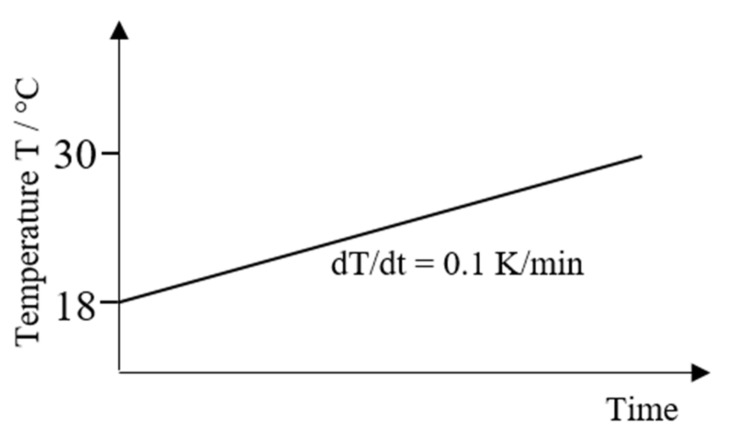
Temperature-time profile for determination of the specific heat capacity cp.

Fillers might have an impact on the thermal properties of a powder composition. Therefore, it is important to determine the specific heat capacity (cp) in dependence of the filler system. A Calvet calorimeter (C80, Setaram Instrumentation, Caluire, France) was used. The sample with a mass of around 8 g was heated from 18 °C to 30 °C at a heating rate of 0.1 K/min. The temperature-time profile of the measurement is shown in Figure 2.

#### 3.2.3. Sample Parts and Measurement

##### Single Layers

**Figure 3:**
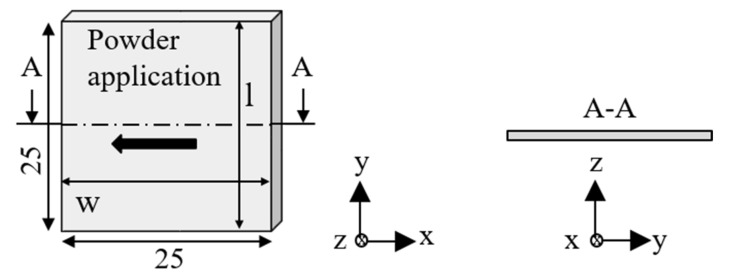
Single layer specimens.

For the evaluation of the energy input from the laser power into the powder bed, quadratic single layers were produced, as can be seen in Figure 3. The length and width with a specified size of 25 mm as well as the layer thickness, which depends on the laser energy density and on the filler content, was measured.

##### Parts

**Figure 4:**
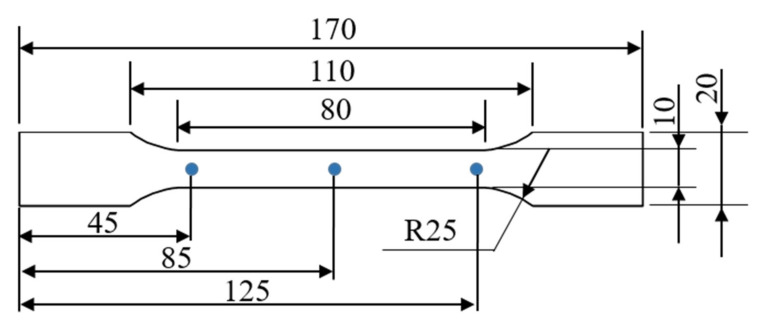
Tensile bar according to DIN EN ISO 3167.

The width of the tensile bars, which were produced according to DIN EN ISO 3167 [20] were measured by a caliper at three different spots (see Figure 4). Mechanical properties of these material systems were published in a former study [5]. The specified width size was 4 mm.

##### Optical Measurement

The dimensions of the single layers were measured optically using a stereo microscope (SteREO Discovery, Carl Zeiss Microscopy Deutschland, Oberkochen, Germany) viewed with transmitted light. In doing so, only the molten areas can be measured, while adhered powder particles can be excluded. Three measuring values were taken per layer.

## 4. Results and Discussion

### 4.1. Powder Properties

#### 4.1.1. Optical Behavior

**Figure 5:**
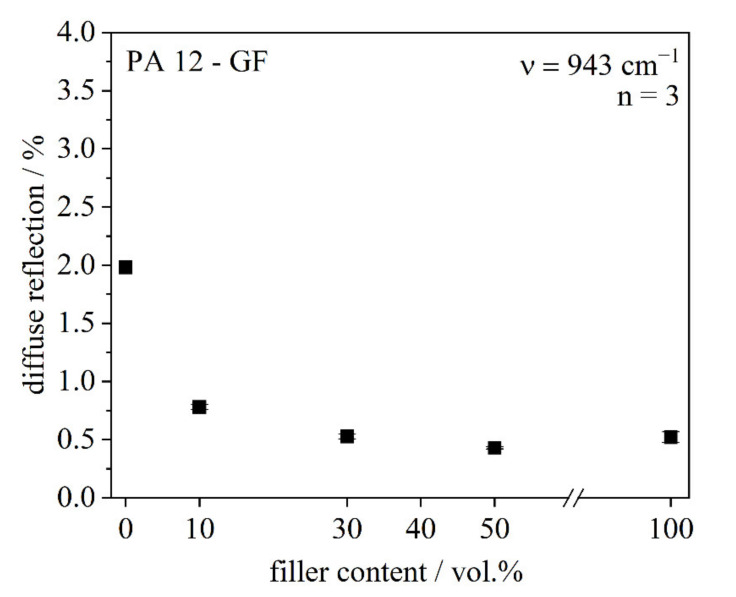
DRIFTS measurements of different PA 12/short glass fiber composites.

The optical behavior of the powder composites is essential for estimating the energy input. The more energy is absorbed, the higher is the amount of molten material. DRIFTS is a method to measure the diffuse reflection of powder materials as infrared light is focused on the sample using an infrared spectrometer. The diffuse reflected beams are then detected. As can be seen in Figure 5, a higher glass fiber content leads to a decrease of the diffuse reflection, which might result in a decreased laser penetration depth. The clearest effect on the diffuse reflection can be observed between 0 and 10 vol.% glass fiber content. By increasing the fiber content to 30 or 50 vol.% the diffuse reflection decreases only a low amount, but not as much as between 0 and 30 vol.%. Thus, the conclusion is that for this effect it is more significant if there are any fillers inside at all than the quantity of fillers. The absorption coefficient is increased by a higher glass fiber content, as there are more interfaces within the system [21]. In this study, the used fillers were short glass fibers. The two terms “filler” and “(short) glass fibers” are used synonymously in this case.

#### 4.1.2. Specific Heat Capacity

**Figure 6:**
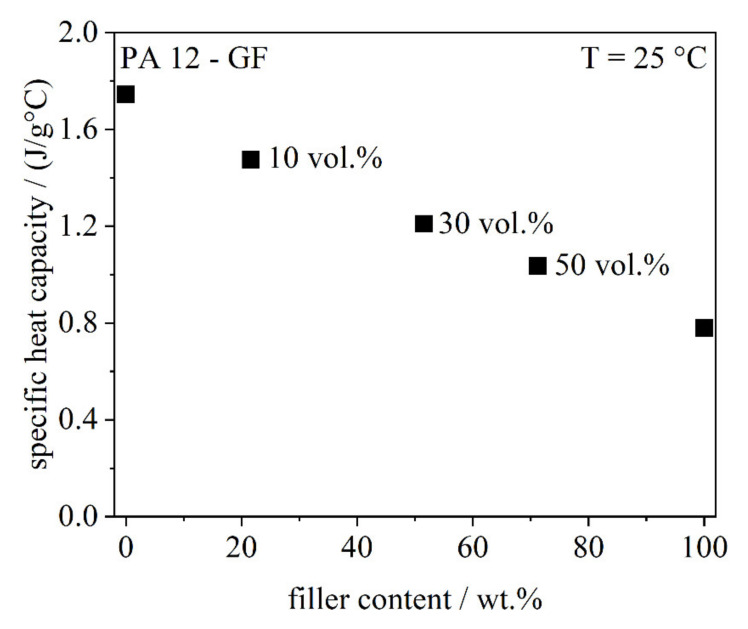
Specific heat capacity in dependence of the filler content.

The specific heat capacity is the quantity of heat that has to be applied to a material to raise its temperature by 1 °C at constant pressure, which describes the capacity of a material to store thermal energy. It is dependent on the temperature, the heat flux and mass [22]. The specific heat capacity is measured using a Calvet calorimeter, where the powder is heated and a sensor, which surrounds the sample completely, measures the heat flow very accurately. By adding fillers, the heat capacity decreases linearly according to the rule of mixture (see Figure 6). The specific heat capacity is plotted versus the filler content in mass fraction as voids occupy a considerable volume in a powder bed and the mass and heat capacity of voids is zero.

A low specific heat capacity leads to a higher temperature diffusivity during melting [23]. Theoretically, if the heat flux and mass is constant, a reduction of heat capacity leads to an increase of the temperature and therefore to an enhanced melting. Thus, compared to the neat material, the thermal household is changed, resulting in an increased partial melting and to potentially enhanced part dimensions, in turn. The role of heat conductivity was not considered in this case and will be considered in future experiments.

### 4.2. Part Dimensions

The changed optical and thermal powder properties due to the increasing filler content lead to altered part dimensions. This is shown on single layers and a transfer on parts will be made.

#### 4.2.1. Single Layer Dimensions

**Figure 7:**
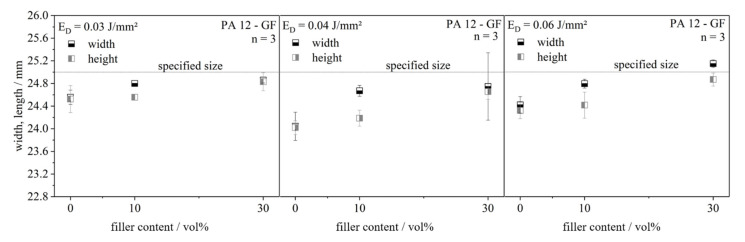
Width and length of single layers in dependence of the filler content at three different energy densities.

**Figure 8:**
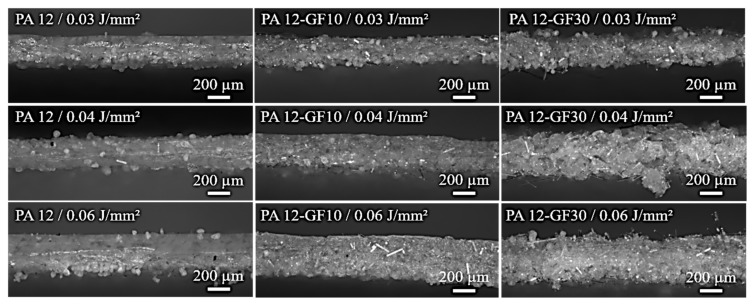
Cross sections of single layers in dependence of the filler content and energy densities.

The effects of increased absorption and decreased specific heat capacity on the dimensions of the single layers are shown in Figure 7 and Figure 8. It becomes obvious that the part dimensions are generally below the specified size and increase with rising filler content. The sample with neat material shows the lowest dimensions due to the highest shrinkage. The build job stops just after exposure of the single layer and the heat leaves the material system. Curling, i.e., bending of the edges, occurs due to crystallization, as only single layers are generated and no isolating layers are applied. The width of the layer tends to be higher than the length. This phenomenon can be explained with the direction of the powder application. The glass fibers are oriented in this direction and the shrinkage perpendicular to the fibers is higher than the longitudinal one. Thus, a deviation from the quadratic shape specification can be observed. With increasing filler content, the specified size can be reached, because a higher filler content minimizes shrinkage. A significant dependency of the part dimensions on energy density cannot be seen.

The optical analysis of the cross-sections of the single layers is depicted in Figure 8. An increasing layer thickness with filler content at the same energy density can be observed. The glass fibers lead to an increased absorption and more energy input into the powder bed as they act as a light conduct. A higher energy density and therefore a higher energy input results in increased part thickness. Moreover, it can be observed that the surface roughness increases with a higher filler content due to the length of the fibers.

**Figure 9:**
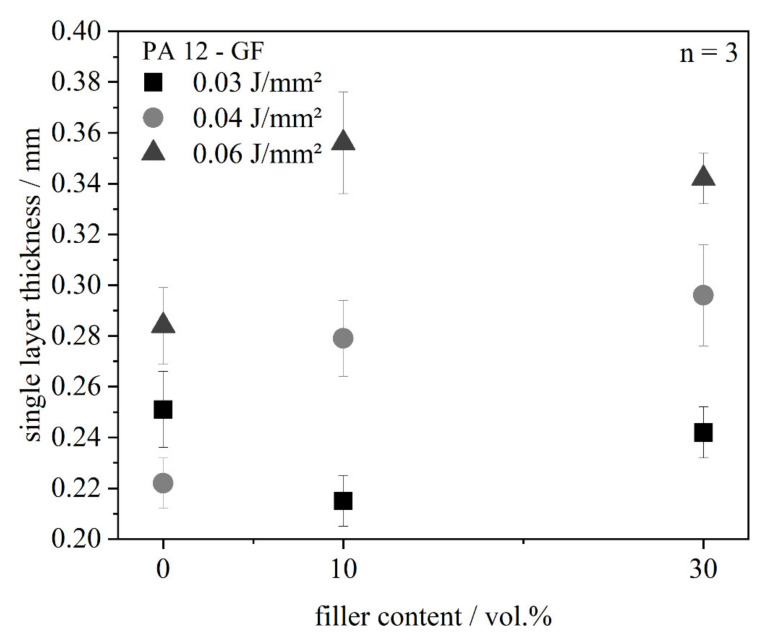
Single layer thickness in dependence of the filler content.

In Figure 9, the thicknesses are depicted metrologically. At an energy density of 0.04 and 0.06 J/mm^2^, an increase of the layer thickness compared to the neat PA 12 material can be seen. The most clearly change can be seen from 0 to 10 vol.% filler content, which is consistent with the measurements of the diffuse reflection. From 10 to 30 vol.% the change lies within the measurement error. It can be summarized that the single layer thickness is increased when the energy density is increased at constant filler content on the one hand, and when the filler content is increased at constant energy density due to absorption behavior and decreased heat capacity on the other hand. Nevertheless, the increase of the layer thickness is not stringent, as loose powder might adhere to the bottom side of the single layer, which can have a clear effect on the measurement of the single layer thickness as well as the cleaning grade of these loose powder particles. Moreover, the surface roughness increases due to the fiber lengths. The filled system consists of the glass fiber and the polymer. When the laser exposes the parts and the polymer powder is transferred into melt, the crystalline areas are decreased and the transmittance is increased, while absorption of the polymer is reduced [18], leading the energy into the depth of the powder bed. At the same time, the glass fibers lead to a higher absorption, which is an additional effect.

An analysis of variance (ANOVA) was made to evaluate if the factors (energy density and filler content) are statistically significant (Table 4). The null hypothesis H_0_ was that there is no difference between the expectancy values of the groups. The confidence level was set to 95%. The values are tested on normal distribution beforehand.

As the *p*-values for the factors ‘energy density’ and ‘filler content’ are both bigger than 0.05, the values were not statistically significant.

In the following, it is analyzed if multilayer parts show a clearer tendency.

#### 4.2.2. Part Dimensions

**Figure 10:**
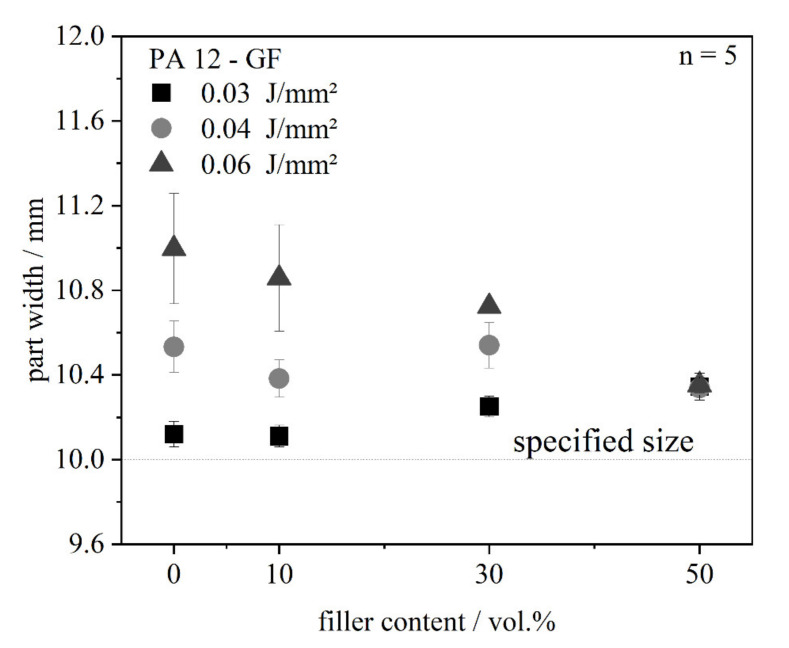
Dimensions of tensile bars in dependence of the filler content at three different energy densities.

It could be shown that the dimensions of the single layers change in dependence of the glass fibers. It was tested if this phenomenon can be transferred to multi-layer parts by measuring the dimensions of tensile bars (Figure 10). No scaling factors are applied to gain basic understanding of the material behavior. The part width of the tensile bars was relatively constant, while the interference can be explained by the used measuring method. A micrometer gauge was used to measure the parts and adhering powder on the part is included, so the maximum values were determined.

**Figure 11:**
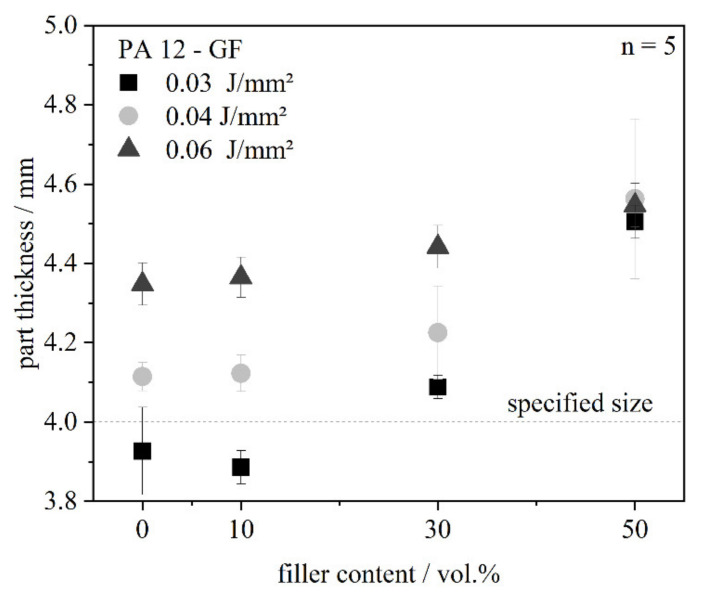
Part thickness of tensile bars in dependence of the filler content at three different energy densities.

Analogous to the single layer, an increase of the part thickness with higher filler content can be seen (Figure 11). Again, this can be explained by the increased absorption and the increased specific heat capacity, which is responsible for the deviation in geometry. It is obvious that the standard deviation enhances at a filler content of 30 and 50 vol.% compared to the low filler content or the neat material, respectively. The fibers with a medium length of around 200 µm result in a higher powder bed roughness following in an increased roughness of the parts, which is also observable in Figure 8. A higher energy density leads to a higher part thickness, because the energy input into the powder bed is increased. It seems that the effect of the energy density gets lower at a higher filler content. At a very high filler content of 50 vol.%, the energy density is irrelevant to the part thickness. This means the filler content is the more dominant factor.

Based on the values in Figure 11, an ANOVA regarding the significant factors for the part thickness is conducted, Table 5.

The *p*-values below 0.05 for the factors ‘energy density’ and ‘filler content’ show that both factors are statistically significant.

Based on these results, new process parameters for filled systems could be derived. It depends which command variables are to be followed. For a higher dimensional accuracy, the energy density can be reduced as the specified size is reached. On the other hand, the mechanical properties at different energy densities have to be considered, which have been published in a former study [5]. While the tensile strength and elongation at break of the filled systems is not dependent on the energy density, the tensile modulus of systems with a higher filler content of 30 vol.% is lower compared to a higher energy density. Therefore, for the production of prototypes with low requirements on mechanical properties, the energy density can be reduced, while the focus for technical parts should be set on mechanical properties.

## 5. Conclusions

In this study, the thermal and optical properties of short glass fiber filled polymer system and their effect on part geometry was analyzed. The diffuse reflection decreased with rising filler content, because at the wavelength of the CO_2_-laser glass exhibits high absorption, and moreover, the number of interfaces is increased. The specific heat capacity, which is also decreased by adding glass fibers, results in an increased partial melting of powder and changes the geometrical dimensions of parts. Analyzing the parts’ geometry in dependence of the energy density and filler content, the following conclusions can be drawn. A deviation from the specific size can be observed on single layers. The orientation of the glass fibers in powder coating direction result in a preference direction of shrinkage and lead to a higher length compared to width. The layer thickness is increased with rising filler content due to higher absorption of the glass content, which could be shown by the decrease of the diffuse reflection and specific heat capacity. Moreover, the surface roughness increases due to the fiber lengths. It has to be taken into account that the powder properties change if fillers are added and therefore processing parameters often have to be adjusted to the material. Furthermore, the transfer from single layers to parts is made. No change in the part width was observed with increasing filler content. Analyzing the part thickness, an exponential increase up to a filler concentration of 50 vol.% occurs. From this perspective, combining high part accuracy and mechanical properties can be made by an adjusted contouring exposure. Less energy density in the edge and a higher energy input in the core area would lead to an increased accuracy as the melt pool is frozen outside, and at the same time enough energy input can be guaranteed in the inside of the parts to reach required mechanical values. In summary, a deeper insight into the material beam interactions regarding glass fiber filled systems could be made, from which it is important to derive an optimum adjustment of processing for filled systems in powder bed fusion.

## Figures and Tables

**Table 1:** Used matrix polymer PA 12.

Polymer	PA 12	REM
type	PA 2200	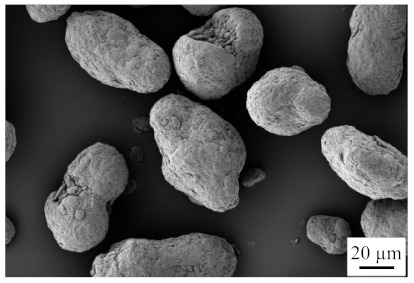
supplier	EOS GmbH	
raw density [g/cm^3^]	1.01	
melting temperature Tm [°C] ^1^ (dT/dt) = 10 K/min	185	
crystallization temperature Tc [°C] ^1^ (dT/dt)= 10 K/min	150	
d_10.3_/d_50.3_/d_90.3_ [µm] ^1^	46/61/83	

^1^ own measurements.

**Table 2:** Used fillers short glass fibers.

Filler	Glass Fiber	REM
type	EMG 10 70	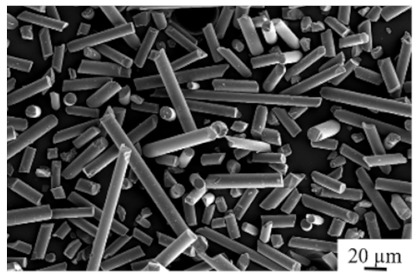
supplier	ECTA Handels-gesellschaft mbH	
raw density [g/cm^3^] ^1^	2.5	
bulk density [g/cm^3^] ^1^	1.96	
Fiber diameter [µm]	10	
d_10.3_/d_50.3_/d_90.3_ [µm] ^1^	21/67/241	

^1^ own measurements.

**Table 3:** Processing parameters of the composites.

PA 12/SGF Vol/Vol.%	T_B_ °C	P_L_ W	v_s_ mm/s	E_D_ J/mm^2^
90/10 70/30 60/40 50/50	168	9.3 16.3 23.9	1900	0.03 0.04 0.06

**Table 4:** ANOVA for values of single layer thickness.

Source	DF	Adj SS	Adj MS	F-Value	*p*-Value
Energy density	2	0.013025	0.006512	6.71	0.053
Filler content	2	0.002742	0.001371	1.41	0.343
Error	4	0.003879	0.000970		
Total	8	0.019646			

(DF: degree of freedom; Adj SS: Adjusted sum of squares, Adj MS: Adjusted mean squares).

**Table 5:** ANOVA for values of part thickness.

Source	DF	Adj SS	Adj MS	F-Value	*p*-Value
Energy density	2	0.20999	0.104993	10.66	0.011
Filler content	3	0.33824	0.112747	11.44	0.007
Error	6	0.05912	0.009853		
Total	11	0.60734			

## Data Availability

The data presented in this study are available on request from the corresponding author.
